# Retraction: Effect of histone deacetylase inhibitors trichostatin A and valproic acid on hair cell regeneration in zebrafish lateral line neuromasts

**DOI:** 10.3389/fncel.2024.1530876

**Published:** 2024-11-25

**Authors:** Frontiers Editorial Office

The publisher retracts the article cited above.

Following publication, concerns were raised regarding the integrity of the images in the published figures. The authors failed to provide a satisfactory explanation during the investigation, which was conducted in accordance with Frontiers' policies.

This retraction was approved by the Chief Editors of Frontiers in Cellular Neuroscience and the Chief Executive Editor of Frontiers. The authors received a communication regarding the retraction and had a chance to respond. This communication has been recorded by the publisher.

